# The Double-Edged Sword of Safety Training for Safety Behavior: The Critical Role of Psychological Factors during COVID-19

**DOI:** 10.3390/ijerph191710951

**Published:** 2022-09-02

**Authors:** Xin Ning, Jiwen Huang, Chunlin Wu, Tong Liu, Chao Wang

**Affiliations:** 1 School of Investment and Construction Management, Dongbei University of Finance and Economics, Dalian 116025, China; 2 School of Economics and Management, Beihang University, Beijing 100191, China; 3 Beijing Key Laboratory of Emergency Support Simulation Technologies for City Operations, Beihang University, Beijing 100191, China; 4 Bert S. Turner Department of Construction Management, Louisiana State University, Baton Rouge, LA 70803, USA

**Keywords:** conservation of resources theory, role overload, safety behavior, safety training, task setbacks

## Abstract

Safety training (ST) is the primary means of avoiding unsafe behaviors, but it has not achieved the expected impact on improving workplace safety because of the high psychological stress it brings to workers. The coronavirus disease 2019 (COVID-19) further threatens workers’ psychological conditions, thereby diminishing the effectiveness of ST. However, the existing literature has mainly laid emphasis on the bright side of ST and neglected examining its impact on safety behavior (SB) from detrimental psychological factors. Drawing from the conservation of resources theory, a novel two-staged model was established to understand how these psychological factors mediate and moderate the association between ST and SB. We incorporated resource consumption (e.g., role overload (RO) and COVID-19-related task setbacks) and resource generation (e.g., psychological resilience) into the model to consider both detrimental and protective psychological factors against ST. We then implemented a time-separated, three-wave data collection on a sample of frontline workers to validate this hypothetical model. Consistent with our hypothesis, RO played a significant mediating role between ST and SB, that is, ST leads to RO, and in turn, holds up SB. Surprisingly, contrary to our hypothesis, COVID-19-related task setbacks weakened the negative and indirect impact of ST on SB via RO. This is one of the first empirical studies to highlight how detrimental psychological factors caused by ST constrict or amplify SB. In practice, the efficacy of ST can be enhanced by cultivating psychological resilience and clarifying employees’ job responsibilities to reduce the ambiguity of roles.

## 1. Introduction

Workplace accidents often occur in various sectors [1,2], most of which are caused by employees’ unsafe behavior rather than unsafe working conditions [3,4]. According to statistics from the Ministry of Emergency Management in China, there were 11,076 workplace accidents and 8870 fatal accidents in the first half of 2022. Although the total number of workplace accidents and fatal accidents decreased in successive years, the topic of workplace safety remains severe and critical [5]. Nevertheless, the US Occupational Safety and Health Administration (OSHA) aims to ensure safe and healthful working conditions for workers [6]. While the marginal rate of the return on investment in hardware facilities is decreasing, accident prevention from the organizational and psychological perspectives has become increasingly prevalent in occupational safety promotion [7,8]. Safety training (ST) is a key occupational health and safety improvement process aimed at reducing accidents [9,10]. Targeted education and training increases workers’ safety knowledge and improves their safety attitudes and behaviors [11] so that they can complete specific tasks safely [12,13].

Although ST has become a mandatory requirement in workplaces [14], the high fatality rate in construction appears difficult to improve [15], which demonstrates the ineffectiveness of current ST activities. Studies have ignored the possibility that mandatory training activities may add to the psychological burden of employees. When an individual is tasked with too many duties, detrimental psychological factors of perceived role overload (RO) and task setbacks will arise spontaneously and can restrain safety performance. Moreover, the COVID-19 crisis, which necessitated new preventive measures, has had far-reaching consequences for various occupations and introduced new challenges to employees’ daily work routines, thus increasing the risk of psychological distress. Increased knowledge of the underlying mechanisms of how ST promotes safety behavior (SB) considering individuals’ psychological levels, especially detrimental psychological factors, will enable us to design effective training programs in the future. In this context, this study seeks to understand how these psychological factors link and modulate the correlation between ST and SB. It may enable us to develop interventions to improve individuals’ SB and to control and prevent workplace accidents with knowledge regarding the intrinsic relationship between ST and SB.

Past research has mostly neglected exploring the relationships between ST and SB from a trainee’s psychological risk perspective. Many studies considered that ST is a vital dimension of safety climate and investigated its role in improving individual safety awareness and preventing unsafe behavior and accidents [16,17,18]. Furthermore, existing empirical studies have often concentrated on the beneficial effects of ST and demonstrated that it can influence SB by improving motivation, knowledge, and attitude [12,19,20]. On the basis of the conservation of resources (COR) theory, potential detrimental psychological factors are introduced (e.g., RO and COVID-19-related task setbacks) to reconsider the process of ST affecting SB. This reconsideration and challenge to previous ST research is divided into the following three aspects.

First, few studies have considered that ST may increase psychological risks for employees. Specifically, employees are required to complete daily production tasks and are compelled to participate in the organization’s ST. They may feel that the job requirements are beyond their abilities or that they are not competent for multiple tasks, which leads to RO [21]. Scholars have confirmed the impact of RO on SB [22]. However, there is no definitive conclusion about whether RO has a favorable or unfavorable effect on occupational safety in the workplace. Moreover, the external environment influences workers’ SB through individual psychological mechanisms [23]. Therefore, we probe into the underlying mechanism of ST on SB and the mediating role of RO between ST and SB.

Second, unique and unforeseen disruptive COVID-19-related task setbacks may be detrimental to the effectiveness of ST. Evidence suggests that the mental health of most people has been negatively impacted by the pandemic [24]. Workplaces are fraught with greater risks than ever. Research has also shown that worker fatigue and distraction may increase unsafe behaviors in the workplace and then cause additional accidents [25]. Pandemic prevention measures, including frequent hand washing, temperature monitoring, and registration in and out of the workplace, are vital to preventing the virus from spreading in a labor-intensive environment. These measures bring changes and uncertainty to daily work practices and may have far-reaching adverse impacts on the psychological states of workers. Compliance with social distancing guidelines and travel bans have inconvenienced people and interrupted their daily work routine. These constraints threaten psychological health and put the completion of work at risk. Therefore, we included COVID-19-related task setbacks [26] in the model, which refers to any issues caused by the pandemic that did not exist before it. These unforeseen and unique COVID-19-related task setbacks may affect the psychological states and behavioral outcomes of workers.

Third, as a reaction to the threat, people are more inclined to adopt preventative measures to reduce their occupational health risks [27]. However, workers are still required to work hard and devote themselves to daily production activities. There is an urgent need for positive strength to help them to complete their work and weather the storm. Psychological resilience [28,29], a protective psychological variable, helps people to maintain a sense of purpose and motivation despite enormous obstacles. When facing difficulties, individuals with various levels of psychological resilience react differently, thus leading to varied SB. Additionally, resilience can be enhanced as an individual resource [30]. Therefore, we introduce psychological resilience to adjust the RO perceived by workers.

Through these reconsiderations, we innovatively shift the predominant focus from the positive effects to the possible psychological risks and hazards caused by ST. To clarify and understand the intrinsic relationship between ST and SB at the individual psychological level, using the COR theory as a theoretical lens, we incorporate both resource consumption (e.g., detrimental psychological factors of RO and COVID-19-related task setbacks) and resource generation (e.g., protective psychological factors of psychological resilience) into the process to examine how ST affects the SB of employees profoundly and holistically.

## 2. Theoretical Background and Hypothesis Development

### 2.1. Theoretical Foundation: Conservation of Resources Theory

The COR theory was proposed by Hobfoll [31] and states that individuals endeavor to acquire, protect, and retain their resources [32], with resources (e.g., energy, knowledge, and support) defined as matters that people value, which can be construed in the light of expenses and benefits [33,34]. Particularly, the COR theory provides a systematic and overall framework for our research and allows us to understand how ST may affect SB while considering both resource-consuming and resource-generating processes.

The COR theory is widely used to explore the role of stress and emotional exhaustion [35,36]. It originally emerged as a stress theory and provides a new perspective for predicting employee behavior. The COR theory suggests that stress may occur when a person perceives that their resources are threatened or has experienced resource loss, and that individuals tend to conserve existing resources to prevent future resource threats or losses. In this study, we assume that mandatory ST activities may consume a vast array of individual resources, leading to high perceived RO. To cope with this subtype of role stress, individuals must use their available physical and psychological resources, which, if depleted, can lead to adverse consequences such as worse safety performance. To complicate matters, task-related setbacks during COVID-19 have adversely affected employees’ psychological states, which may also result in significant resource loss. Our study focuses on detrimental psychological factors to reflect resource consumption in the process of ST affecting SB. According to a key tenet of the COR theory, losing resources hurts individuals psychologically more than the benefits after gaining these resources [32]. Thus, individuals can take measures to preserve the remainder. The COR theory provides a useful tool for understanding resilience [37]. Resilience is defined as an individual resource to handle or overcome various adversities and perceived stress [38]. Research has increasingly focused on how resilience may alter mechanisms by which stressful experiences negatively affect psychological adverse outcomes [39]. Consequently, psychological resilience may act as a key protective psychological factor to modify resource depletion and allow people to cope with stress. Drawing on both resource-consuming and resource-generating processes, we explore the role of detrimental and protective psychological factors based on the COR theory.

### 2.2. The Mediating Function of Role Overload

RO occurs when job demands surpass the available resources [40]. One individual is often tasked with several duties in such a workplace. When workers believe there is a gap between their abilities and the expectations imposed on them, perceived RO will naturally arise. ST means the safety instructions and guidance that employees receive on the job. It is deemed as an effective means to facilitate employee compliance and reduce injuries [41]. According to the COR theory [42], workers who devote themselves to ST may deplete a vast array of resources, which makes it difficult for them to implement multiple tasks simultaneously. The sense of losing job control in one’s workplace may cause mental and physical stress for workers [43]. On-site stressors are an increasing concern for employees and managers [44,45]. We hypothesize that ST may serve as an environment-related stressor, thus causing workers to perceive RO.

**Hypothesis** **1.** *ST is positively associated with RO.*

The meta-analysis of previous studies finds that RO has a positive relationship with job stress, burnout, and turnover intention, while being negatively correlated with organizational commitment [46,47]. RO causes individuals to devote their efforts, energy, and resources to cope with excessive job demands, which affects their job engagement and reduces performance [48]. Moreover, evidence shows that role stress is negatively related to SB [49]. Employees who experience long-term RO may overdraw their physical and psychological resources to complete excessive task requirements [42]. RO may cause employees to fail to activate the compensation mechanism for resource loss, thus forming a “depletion spiral” of individual resources [48]. When RO is high, employees may have elevated levels of anxiety and dissatisfaction with work and life, which manifests as a lack of energy and capacity to commit themselves to work and a greater skepticism or denial of the organization’s arrangements for safety. Thus, it is harmful to workers’ psychological health and undermines SB performance levels. RO is a concept originating from the job demands–resources theory [44,50,51]. We use the concept of job hindrance to explain the relationship between RO and SB [52]. During COVID-19, frontline workers working in densely populated workplaces have been forced to implement onerous pandemic precautions. They perceive RO as a job hindrance and, therefore, are reluctant to complete specific tasks cautiously and safely. When individuals see the stressor as a hindrance rather than a challenge, it may boost negative emotions (such as disappointment and diffidence) and encourage individuals to take inappropriate measures, thus leading to worse performance.

**Hypothesis** **2.** *RO is inversely associated with SB*.

RO is affected by ST and impacts an individual’s SB. The theory of social cognition [53] indicates that human activities are interactively determined by three factors: individual behavior, psychological cognitive processes, and the external environment. Individual behavior is the result of external environment and internal detrimental psychological factors. Therefore, we expect that RO, an expression of individual internal psychological characteristics, acts as a mediator between ST and SB.

**Hypothesis** **3.** *RO mediates the relationship between ST and SB*.

### 2.3. The Moderating Role of COVID-19-Related Task Setbacks

The Centers for Disease Control and Prevention (CDC) [54] has recommended several measures to keep COVID-19 from spreading, e.g., washing hands frequently, wearing masks, implementing temperature monitoring, registration when entering and leaving workplaces, maintaining a safe social distance with others at work, and a combination of preparatory work and an isolation policy after resuming work. Although these time-consuming measures have played a certain protective role, they have disturbed the work routines and psychological states of workers. Based on the concept of goal disruption [55] and organizational constraints [56], Chong [26] defined COVID-19-related task setbacks as the task-related disruptions and inhibitions emerging from the pandemic situation. These may involve COVID-19 itself, related issues such as lockdown regulations and social distancing, or any issues that have arisen that did not exist before the pandemic. In light of the COR theory [42], COVID-19-related task setbacks consume significant emotional and physical resources and force individuals to respond adaptively. Workers in high-risk workplaces must adhere to the regulations recommended by the CDC and manage setbacks created by the pandemic, all while performing the original labor activities. To maintain the further loss of limited individual resources, workers often adopt negative behaviors such as avoidance, reducing effort, and other strategies to maintain their emotional resources. Therefore, COVID-19-related task setbacks may cause fluctuations in psychological pressure. After receiving the same ST, people with a higher perception of COVID-19-related task setbacks may experience greater RO. Consequently, the SB of workers will be affected. Thus, we posit that:

**Hypothesis** **4.** *COVID-19-related task setbacks moderate the positive and direct relationship between ST and RO such that this relationship is more positive with increased COVID-19-related task setbacks than with decreased COVID-19-related task setbacks*.

**Hypothesis** **5.** *COVID-19-related task setbacks moderate the positive and indirect relationship between ST and SB (via RO) such that the indirect relationship will be less positive with increased COVID-19-related task setbacks than with decreased COVID-19-related task setbacks*.

### 2.4. The Moderating Role of Psychological Resilience

Resilience originates from physical science, but psychological resilience, as a personality characteristic, means the capacity of recovering quickly from adversity in the face of various difficulties and challenges [57]. Divergences in individual characteristics will lead to them perceiving work pressures differently even when they are in the same working environment and facing the same workload as others, which will ultimately affect their safety performance [58]. Psychological resilience is defined as a dynamic process of positive adaptation under the circumstances of adversity, trauma, tragedy, and threats [59] (the American Psychological Association, 2012). Psychological resilience has a positive influence on individuals’ perception and response to stress in a difficult work environment [38]. Resilience enables people to tolerate a large amount of work, thus reducing the likelihood of running out of personal resources to perform their duties [60]. Rushton [61] found that resilience can also protect employees from emotional exhaustion.

When faced with the same situation and burden, some employees can manage it calmly without arrogance or rashness, while others feel restless. Employees with resilience may believe that they have adequate resources to manage the workload. Consequently, they are more likely to focus on their tasks without anxiety and can effectively cope with setbacks with extraordinary resilience. However, those with low resilience find it more difficult to manage negative feelings or distracting moods caused by stress [62]. The psychological resilience of frontline workers affects their perceived stress, resulting in divergent SB. Yet, few empirical studies have examined the protective role of psychological resilience on SB in high-risk industries [63]. Therefore, we explore the moderating role of psychological resilience.

**Hypothesis** **6.** *Psychological resilience moderates the negative and direct relationship between RO and SB such that this relation is less negative (or even positive) at higher psychological resilience than at lower psychological resilience*.

**Hypothesis** **7.** *Psychological resilience moderates the positive and indirect relationship between ST and SB (via RO) such that the indirect relationship will be more positive at higher psychological resilience than at lower psychological resilience*.

**Figure 1:**
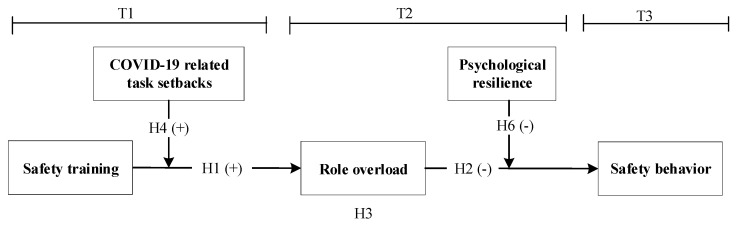
The proposed conceptual model.

We then propose the conceptual model in our study (see Figure 1). T1, T2, and T3 represent three different phases of analysis, spaced apart by 21-day intervals.

## 3. Methodology

### 3.1. Participants and Procedure

We selected construction workers as an example because the workplaces of the construction industry are places with significant risk of injury or death [2]. We commenced by distributing preliminary questionnaires to workers from Chinese construction sites in Henan Province for a pre-investigation. Based on the feedback, we revised the questionnaire to clarify the wording. We launched the official inquiry in March 2021. Our target population mainly included construction workers from four provinces across China (Guangdong, Zhejiang, Hubei, and Henan Province) undergoing compulsory ST provided by their employers. We collected data at three time-points (i.e., T1, T2, and T3) in this study. Construction site environments are characterized by rapid changes and lengthy time span, in which the respondents may not guarantee their participation in all three rounds of surveys. Therefore, we conducted surveys with a 21-day interval after referring to previous studies [64,65]. Participants assessed ST and COVID-19-related task setbacks through the first self-report survey (T1). The ST interventions concerned safety-related knowledge, skills, and awareness. We assumed that the utility of the training may be affected by several psychological factors. Therefore, we measured the perceptions of RO and psychological resilience 21 days after training at T2. To validate the effectiveness of training, we conducted the third self-report survey at T3 to measure the SB of workers. We received 431 valid responses in T1. Further, we proceeded to send questionnaires to these 431 workers. In T2, 398 individuals participated; 33 individuals were not eligible (resigned from the construction site = 13; did not want to participate = 20). In T3, 367 individuals participated with a final valid response rate of 85.1%. Overall, 81.7% (n = 300) of workers were male, while the rest were female. The workers were mostly between the ages of 21 and 50. In terms of work experience, 76.8% of the workers (n = 282) had been in their current jobs for more than five years. Many possessed lower-level diplomas, whereas 21.6% (n = 79) possessed at least a bachelor’s degree.

### 3.2. Questionnaire Design

We used a 5-point Likert scale ranging from 1 (strongly disagree) to 5 (strongly agree) to assess the questions. We used the mean value to calculate the scale score for all multi-item scales.

#### 3.2.1. Safety Training (T1)

Adapted from Neal et al. [66], Vredenburgh [67], Flin et al. [68], and Lu [69], this notion was measured by six items. Example items include “The company provides comprehensive and adequate safety training” and “The safety training program can help to enhance my safety knowledge.”

#### 3.2.2. COVID-19-Related Task Setbacks (T1)

Chong [26] modified the adapted disruptive work events scale [55] to three items in the context of COVID-19. A sample item is “Today, something related to the COVID-19 situation disrupted me from my planned work goals.” A high score indicates that task setbacks caused by the pandemic have a strong influence on an individual.

#### 3.2.3. Role Overload (T2)

We adopted a total of four measuring items from the studies of Schaubroeck et al. [70] and Beehr et al. [71]. An example item is “I have to do too many things at work”. A high score suggests that the individual perceives significant RO.

#### 3.2.4. Psychological Resilience (T2)

We assessed psychological resilience using a six-item scale created by Luthans [72] which evaluates an individual’s tolerance for negative influences and capacity to actively accept change. High scores represent a person’s capacity for proactive adaptation to change.

#### 3.2.5. Safety Behavior (T3)

We adopted the measurement items (seven items for compliance behavior and six for participation behavior) for SB from Griffin and Neal [73]. Two examples are “I utilize all essential safety equipment to accomplish my job” (compliance behavior) and “I go above and beyond to promote workplace safety” (participation behavior). A high score indicates that the participant is inclined to perform SBs.

### 3.3. Analysis Strategy

Before the formal survey, we analyzed the 127 effective pre-survey questionnaires and the results showed that the questionnaires had high reliability (Cronbach’s alphas = 0.976, F-statistic = 65.447, *p* < 0.01). As an indicator of the dependability of internal consistency, the threshold of Cronbach’s alpha is 0.70 [74]. We conducted a three-wave data analysis. First, we undertook descriptive analysis and reliability tests. Second, we performed confirmatory factor analyses (CFA) on our multi-item scales to determine convergent and discriminant validity and compute the indices to verify convergent validity. Additionally, the model fit was estimated using the maximum likelihood estimation. Finally, hypotheses were tested utilizing path analyses and moderated mediation analyses. The direct effects (H1 and H2), mediating effects (H3), and moderating effects (H4–H7) were tested sequentially. For the mediation and moderation analyses, we used the PROCESS macro of SPSS to construct 5000 bootstrap samples and 95% confidence intervals (CIs) with the bootstrapping technique [75,76]. It is noted that if a 95% bias-corrected CI excludes zero, the effect is significant [77].

## 4. Results

### 4.1. Reliability and Validity

In Table 1, we present descriptive statistics and correlations among variables. The Cronbach’s alpha values are all above 0.7 [74], which indicates high reliability. The results in Table 1 show that ST is significantly correlated with SB (r = 0.548, *p* < 0.01) and RO is correlated with ST (r = 0.235, *p* < 0.01) and SB (r = −0.280, *p* < 0.01). Thus, the correlation coefficient between each variable is significant and appropriate. For the controls, RO is related to work experience (r = 0.117, *p* < 0.05). However, no significant correlation is shown between other controls and the focal variables.

We then conducted CFA to test validity. Compared to alternative models, our hypothesized five-factor baseline model provided substantial improvement in fit indexes (see Table 2). Thus, our measurement model shows good structure validity.

We examined convergent validity by calculating standardized factor loading (SFL), composite reliability (CR), and average variance extracted (AVE) of the five focal variables. All SFL values in our analysis to demonstrate convergent validity range (0.601–0.986) were above the 0.50 value proposed by Hair et al. [78]. The range of CRs (0.889–0.989) was significantly higher than Fornell and Larcker’s critical threshold of 0.60 [79]. The range of AVE values (0. 729–0.959) exceeded the 0.50 value from Fornell and Larcker [79], indicating excellent explanatory power. The convergent validity of our measurements is, therefore, confirmed. The square root of AVE exceeded all correlations with any other variables in the measurement model, thus suggesting high discriminant validity. All indicators were statistically significant (*p* < 0.05), which suggests a well-structured measurement model [79]. The results indicate that our measurement model has a five-factor structure with high criterion-related convergent and discriminant validity.

### 4.2. Hypotheses Testing

We examined the moderated mediation model using PROCESS [75]. In Table 3, ST had a significantly positive effect on RO (B = 0.404, SE = 0.128, *p* < 0.01). RO had a significantly negative relationship with SB (B = −0.694, SE = 0.093, *p* < 0.001). Thus, Hypothesis 1 and 2 are supported.

We then examined the mediating role of RO using the bootstrapping method [75,76]. We used the indirect approach and replaced 5000 bootstrap samples from the total samples [80]. The indirect effect of ST on SB via RO was −0.024 (SE = 0.011, *p* < 0.001, 95% CI = [−0.042, −0.006]), which is significant as the CI excludes zero. Thus, Hypothesis 3 is supported.

**Figure 2:**
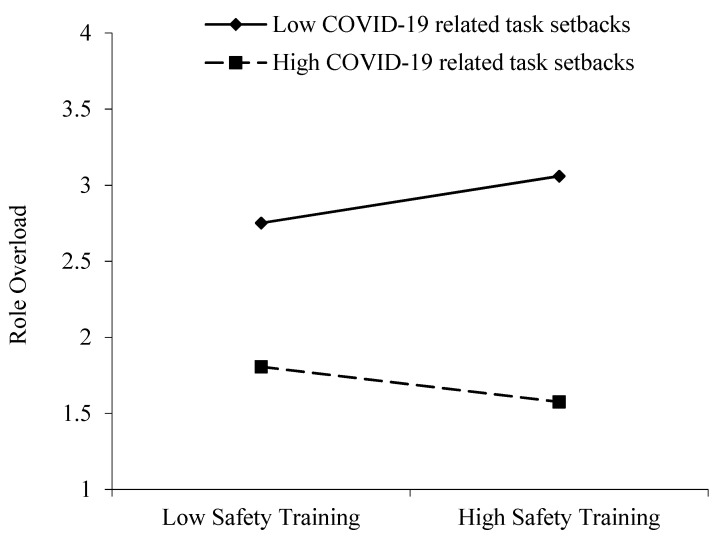
Moderating effect of COVID-19-related task setbacks on the relationship between safety training and role overload.

**Figure 3:**
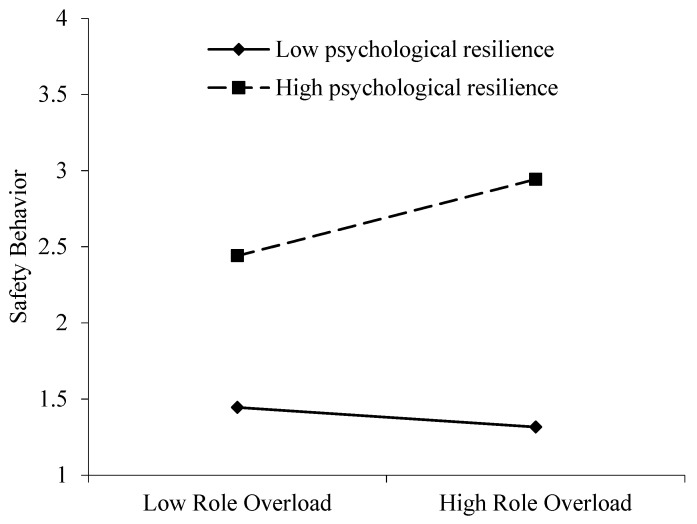
Moderating effect of psychological resilience on the relationship between role overload and safety behavior.

For the direct moderating effects, in Table 3, we found that the interaction of ST and COVID-19-related task setbacks had a significant effect on RO (B = −0.101, SE = 0.046, *p* < 0.05), and the interaction of RO and psychological resilience had a significant effect on SB (B = −0.143, SE = 0.020, *p* < 0.001). To illustrate how each moderator affects the link between variables, the conditional effects for low (−1 SD) and high (+1 SD) levels were examined. ST had a significantly negative effect on RO for high levels of COVID-19 task setbacks (B = −0.089, SE = 0.130, *p* < 0.05, 95% CI = [−0.168, −0.010]) and was not related for low levels of COVID-19 task setbacks (B = 0.174, SE =0.063, *p* > 0.05, 95% CI = [−0.051, 0.399]). Hypothesis 4 is partially supported. We expected a more positive relationship between ST and RO for higher levels of COVID-19-related task setbacks. Surprisingly, Figure 2 illustrates how COVID-19-related task setbacks mitigated the effect of ST on RO. Figure 3 shows that RO had a significantly positive relationship with SB for high levels of psychological resilience (B = 0.192, SE = 0.030, *p* < 0.05, 95% CI = [0.133, 0.250]), and RO had no significant relationship with SB for low levels of psychological resilience (B = −0.021, SE = 0.023, *p* > 0.05, 95% CI = [−0.067, 0.024]). Thus, Hypothesis 6 is supported. The regression slopes are presented correspondingly in Figure 2 and Figure 3.

We then tested the indirect moderating effects. As shown in Table 4, we found no significant difference between high and low COVID-19-related task setbacks (difference = 0.023, 95% CI = [0.000, 0.046]), which indicated that the indirect effect of ST on SB via RO was not significantly moderated by the COVID-19-related task setbacks. Thus, Hypothesis 5 is unsupported. The effect of ST on SB via RO was 0.055 (SE = 0.023, *p* < 0.05, 95% CI = [0.012, 0.099]) at high levels and was −0.006 (SE = 0.006, *p* > 0.05, 95% CI = [−0.018, 0.005]) at low levels of psychological resilience as a moderator. Bootstrapping results revealed that there is a significant positive indirect effect at high levels but not at low levels of psychological resilience. Additionally, we found a significant difference in indirect effects (difference = 0.061, 95% CI = [0.016, 0.106]). In other words, psychological resilience improved the favorable effect of ST on SB via RO. Thus, Hypothesis 7 is supported.

In summary, the testing results for the hypotheses suggest that although ST activities can improve the SB of workers to some extent, they also place significantly negative psychological burdens (e.g., RO) on them. Workers who perceive RO are less likely to complete specific tasks, especially those associated with behavioral safety. Psychological resilience can mitigate the adverse effects of RO on the SB of workers. Surprisingly, contrary to our initial hypothesis, COVID-19-related task setbacks can weaken the positive association between ST and RO. Considering the moderating effect of psychological resilience, we believed COVID-19-related task setbacks would have had favorable effects on SB.

## 5. Discussion

We constructed a moderated mediation model to explore how ST influences SB through detrimental and protective psychological factors during the COVID-19 crisis. In the model, we examined RO as the mediator and COVID-19-related task setbacks and psychological resilience as the moderators. The COR theory provides us with a new perspective for understanding the dynamic resource changes in the process. ST is significantly and positively related to RO. COVID-19-related task setbacks alleviate the impact of ST on RO and are favorable for improving SB. Moreover, RO has a significant negative association with SB, and psychological resilience acting as the moderator can weaken the impact of RO on SB. In Table 5, we summarize the results.

### 5.1. Theoretical Implications

Our research makes several theoretical contributions. First, traditional research has predominantly considered ST positively and ignored the adverse psychological conditions it creates that jeopardize he SB of workers. Our study challenges this perspective by considering both detrimental and protective psychological factors and how they constrict or amplify the effect of ST. To our knowledge, this is one of the first studies to test how various psychological factors mediate and moderate the relationship between ST and SB empirically, especially during the COVID-19 crisis. Through the lens of the COR theory, we provide a holistic interpretation between ST and SB with the manipulation of psychological factors achieved by synthesizing resource-consuming and resource-generating processes in safety risk management. Our conceptual model emphasizes the resource-consuming mechanism by considering RO as the mediating variable and COVID-19-related task setbacks as the moderating variable. The psychological risk factors and threats in ST affecting SB may consume personal resources. Meanwhile, when viewed as an individual resource that can be enhanced, resilience plays a protective role against resource losses in the process, which is in line with the basic tenet of the COR theory. This study theoretically consolidates the importance of psychological factors in ST and enables us to develop more effective interventions to improve individuals’ SB and to control and prevent workplace accidents.

Second, we advance the existing body of knowledge on the relationship between ST and SB by bringing in the new perspective of employees’ psychological responses with consideration of the employees’ RO. While the important effect of ST on SB has already been pointed out, much of the evidence has been anecdotal or based on managers’ perceptions. This study provides theoretical support that RO plays a potentially important part in the relationship between ST and SB. The COR theory is introduced to interpret the positive relationship between ST and RO (H1). This sheds new light on ST research in the field of accident prevention. Our study also investigates how RO influences occupational SB (H2) by applying the job demands–resources perspective. Past research has been equivocal about whether the role of RO is favorable or unfavorable for safety performance [51,81,82,83], which brought uncertainty about occupation safety and health management in the workplace. To solve this equivocality, we innovatively consider the mediating part of RO (H3) between ST and SB to reveal it as a job hindrance and, thus, contribute to a deeper understanding and explanation of RO’s negative impact on frontline workers’ SB during the COVID-19 pandemic. Our findings confirm that workers who perceive RO may feel more restrained and then consciously decrease their level of safe behavior.

Third, we offer a novel and distinct explanation for COVID-19-related task setbacks to mitigate adverse effects of ST on RO, that is, when COVID-19-related task setbacks increased, the impact of ST on RO was not worsened but alleviated. The concept of COVID-19-related task setbacks was integrated from goal disruption [55] and organizational constraints [56]. Organizational constraints have been categorized as hindrance stressors in the workplace [51,81,82,83]. The majority of research studies conducted in this area state that detrimental psychological factors tend to trigger adverse job performance, which is consistent with our hypothesis. Surprisingly, the empirical result is contrary to our hypothesis and most research, which can be explained by the COR theory. When employees are exposed to pressures, their investment (in terms of energy and time) in their occupation decreases [58]. Owing to energy or time limitations, people may focus only on completing the required job, while ignoring tasks that are beyond their abilities. This result is consistent with the finding of perceived RO reduction. It is also stated that individuals dedicate themselves to mitigating resource losses and acquiring new resources by participating in investment activities [42]. Liu [84] used the COR theory to explain the motivational path of negative emotions in helping behavior in the workplace. Helping behavior is “voluntarily helping others with, or preventing the occurrence of, work-related problems” (Ahmed et al., 2019) [85]. For example, a person may conduct helping behavior to obtain resources through reciprocity [86]. The resources are not necessarily physical but can be psychological (such as optimism and feeling of value to others) or social (such as friendship and seeking help from others) [86]. Since construction workers work and live with colleagues in a relatively closed environment, relationships among team members are close. Motivated by the authentic intention to help the organization achieve goals, workers may help co-workers trapped in COVID-19-related task setbacks. The individual’s perceived RO will be reduced accordingly. Here, our study provides a different perspective to recognize task setbacks. In other words, COVID-19-related task setbacks can weaken the connection between ST and RO, which is consistent with the experimental result.

Finally, this study provides substance to research and practice for occupational safety by introducing psychological resilience as a protective psychological factor against stress (e.g., RO). Bardoel et al. [37] considered psychological resilience to be a significant resource for people. However, there are individual differences in how employees perceive stress [87]. When facing difficulties or adversities, psychological resilience is a coping strategy. Investigating how psychological resilience can help reduce the effect of RO on individuals’ SB is of great value. Additionally, the interaction of RO and psychological resilience has been mostly neglected in previous studies of SB. This paper fills this research gap to a certain extent by considering the interaction of RO and psychological resilience. Our findings show that psychological resilience contributes to improved job performance and spontaneously attenuates the influence of RO on SB. Employees with high resilience may mitigate or eliminate more workplace stress than those with low resilience. Psychological resilience enables employees to adjust to new work situations and handle RO, thus mitigating the negative influence of RO on safety outcomes.

### 5.2. Practical Implications

This research holds meaningful implications for practice. First, effective ST can help workers to have a good grasp of safety knowledge and skills, enable them to identify dangers in the workplace, and help eliminate unsafe behaviors. Therefore, managers should take measures to improve the level of ST in the workplace. Possible measures include focusing on the characteristics of frontline workers and their psychological conditions that make it difficult for them to accept theoretical knowledge and innovating new methods to improve the efficiency and quality of education and training. For example, introducing animation-style video teaching materials can be lively and effective. This method deeply roots safety knowledge in the hearts of the people; the use of dynamic, real, and simulated visualized operation demonstrations is used to comprehensively promote experiential safety education. Therefore, a good level of safety education and training is an important part of safe workplace production in the pandemic.

To prevent RO from becoming an antecedent hindrance that undermines employee SB, managers should clarify each employee’s job responsibilities to avoid putting them in a bad psychological state of work overload. Managers should set clear work goals for workers, enabling them to understand the roles, tasks, and rewards. Open communication channels can also be set up to allow employees to reflect on their real stress to eliminate job hindrances caused by RO. Considering the duality of RO, management structures should investigate the link between role pressure and SB before deciding whether to enhance or decrease employee role pressure. By establishing clearer goals, expectations, and evaluation criteria, the ambiguity of roles can be reduced.

Additionally, management should be committed to promoting the mental health of employees and cultivating their psychological resilience by attending to the buffering effect of psychological resilience, which enhances the positive impact of ST on role pressure and SB. Workers in harsh environments need to keep calm during work, consciously abide by safety regulations, and complete tasks safely. According to this study’s empirical research, high resilience reduces the impact of negative emotions on safety performance for construction workers. To increase workers’ resilience, management should pay attention to the doubts they have experienced in practice, the reflections and breakthroughs experienced after the event, and hold experience-sharing activities. These activities not only help workers learn from local and temporary mistakes, but also allow them to take criticism more openly, thereby realizing occupational safety, health, and well-being.

### 5.3. Limitations and Future Research

Our research has several potential limitations. First, this study relies on a single questionnaire with self-reported data, causing common method bias. To alleviate common method variance, we suggest that future investigations collect data from multiple sources (e.g., from the objective perspectives of managers to evaluate workers’ SB) to verify the relationships revealed in our research. Second, this study proves the specific influencing mechanism of ST on behavior via RO, which warrants future investigation. Further research should be conducted to produce an integrated multi-factor model that incorporates additional organizational (e.g., safety climate) and individual indicators (e.g., safety leadership). Third, we proved the possibility of RO serving as a job hindrance in a certain work context. Future research should attempt to probe the potential effect of RO on safety performance. Furthermore, besides the moderators and mediators in our study, there may be motivational, behavioral, cognitive, and physiological adjustment factors that can influence specific stressor-stress-behavior relationships, such as emotional tension and job dissatisfaction.

## 6. Conclusions

Our research demonstrates the intrinsic mechanism between ST and SB with detrimental and protective psychological factors based on the COR theory. Notably, it shifts the focus of previous literature from positive effects to the possible psychological risks and hazards caused by ST. Results indicate that ST negatively affects SB via RO. Furthermore, RO serves as a job hindrance in this process. Additionally, COVID-19-related task setbacks can weaken the positive effect of ST on RO, and psychological resilience alleviates the negative effect of RO on SB. This study contributes to a theoretical account of the inherent mechanism of SB by emphasizing the psychological factors which can be used to effectively improve occupational safety. Based on these findings, we recommend that managers provide psychological health services to monitor workers’ RO and promote their psychological resilience to prevent possible psychological risk factors from diminishing the effectiveness of ST on SB improvement.

## Figures and Tables

**Table 1:** Descriptive statistics and correlations among variables.

Variables	M	SD	Correlations									
			1	2	3	4	5	6	7	8	9	10
1. Gender (T1)	1.180	0.387	1									
2. Age (T1)	3.170	0.949	−0.069	1								
3. Education (T1)	3.650	0.936	0.184 **	−0.377 **	1							
4. Work position (T1)	1.400	0.593	0.051	0.182 **	0.258 **	1						
5. Work experience (T1)	2.460	1.280	−0.101	0.595 **	−0.012	0.269 **	1					
6. SB (T3)	4.609	0.937	−0.019	−0.015	0.050	−0.018	0.005	(0.989)				
7. ST (T1)	4.521	0.996	0.011	−0.005	0.056	−0.032	0.003	0.548 **	(0.982)			
8. RO (T2)	3.610	1.105	−0.013	0.043	0.064	0.095	0.117 *	−0.280 **	0.235 **	(0.877)		
9. TS (T1)	3.588	1.303	0.035	0.057	−0.060	−0.044	0.029	0.346 **	0.363 **	0.335 **	(0.869)	
10. PR (T2)	4.460	0.967	−0.043	0.047	0.038	0.014	0.067	0.550 **	0.508 **	0.285 **	0.391 **	(0.971)

N = 367. M = means, SD = standard deviations. SB = safety behavior, ST = safety training, RO = role overload, TS = COVID-19-related task setbacks, PR = psychological resilience. Cronbach’s alphas are in parentheses along the diagonal. * *p* < 0.05, ** *p* < 0.01.

**Table 2:** Results of confirmatory factor analysis.

Models	Variable Combination Approaches	*χ* ^2^	df	*χ*^2^/df	CFI	TLI	SRMR	RMSEA
Five-factor model	ST, RO, TS, PR, SB	738.716 *	242	3.053	0.915	0.903	0.062	0.079
Four-factor model	ST, RO, TS + PR, SB	955.244 *	246	4.017	0.878	0.863	0.086	0.094
Three-factor model	ST, RO + TS + PR, SB	1139.386 *	249	4.576	0.847	0.830	0.091	0.104
Two-factor model	ST + RO + TS + PR, SB	1680.240 *	251	6.694	0.754	0.730	0.101	0.132
One-factor model	ST + RO + TS + PR + SB	1902.569 *	252	7.550	0.716	0.689	0.094	0.141

CFI = comparative fit index, TLI = Tucker–Lewis index, SRMR = standardized root mean square residual, RMSEA = root mean square error of approximation. SB = safety behavior, ST = safety training, RO = role overload, TS = COVID-19-related task setbacks, PR = psychological resilience. * *p* < 0.05.

**Table 3:** Results of the moderated mediation model.

Predictors	RO	SB
	B (SE)	B (SE)
Independent variable		
ST	0.404 (0.128) **	0.419 (0.039) ***
Mediating variable		
RO	—	−0.694 (0.093) ***
Moderating variable		
TS	0.724 (0.220) **	—
PR	—	0.836 (0.067) ***
Interactive effects		
ST × TS	−0.101 (0.046) *	—
RO × PR	—	−0.143 (0.020) ***
Control variable		
Age	−0.031 (0.087)	−0.052 (0.034)
Education level	0.028 (0.074)	−0.014 (0.029)
Position	0.190 (0.107)	−0.010 (0.042)
Work experience	0.073 (0.059)	0.025 (0.023)
Constant	0.424 (0.668)	−1.010 (0.301) ***
Model summary	R^2^ = 0.170	R^2^ = 0.814
	F = 9.387 ***	F = 174.726 ***

N = 367. SB = safety behavior, ST = safety training, RO = role overload, TS = COVID-19-related task setbacks, PR = psychological resilience. * *p* < 0.05, ** *p* < 0.01, *** *p* < 0.001.

**Table 4:** Summary of indirect effects.

Conditions	Effects	*SE*	Boot LLCI	Boot ULCI
Moderating role of COVID-19-related task setbacks with CIs				
Indirect paths: ST→RO→SB (corresponding to H5)				
High TS (+1 *SD*)	0.015 *	0.007	0.002	0.031
Low TS (−1 *SD*)	−0.008	0.010	−0.027	0.014
Difference	0.023	0.012	0.000	0.046
Moderating role of psychological resilience with CIs				
Indirect paths: ST→RO→SB (corresponding to H7)				
High PR (+1 *SD*)	0.055 *	0.023	0.012	0.099
Low PR (−1 *SD*)	−0.006	0.006	−0.018	0.005
Difference	0.061 *	0.024	0.016	0.106

N = 367. Bootstrap sample size = 5000. CIs = confidence intervals; LLCI = 95% bias-corrected lower limit confidence interval; ULCI = 95% bias-corrected upper limit confidence interval. SB = safety behavior, ST = safety training, RO = role overload. * *p* < 0.05.

**Table 5:** Summary of results.

Codes	Model Hypotheses	Results	Implications
H1	ST is positively associated with RO.	Supported	ST increases RO.
H2	RO is inversely associated with SB.	Supported	RO could hold up SB.
H3	RO mediates the relationship between ST and SB.	Supported	ST can predict SB via RO.
H4	COVID-19-related task setbacks moderate the positive and direct relationship between ST and RO such that this relationship is more positive at higher COVID-19-related task setbacks than at lower COVID-19-related task setbacks.	Partially supported	The moderating effect is significant, and COVID-19-related task setbacks can alleviate the unfavorable impact of ST on RO.
H5	COVID-19-related task setbacks moderate the positive and indirect relationship between ST and SB (via RO) such that the indirect relationship will be less positive at higher COVID-19-related task setbacks than at lower COVID-19-related task setbacks.	Unsupported	The indirect effect of ST on SB through RO was not significantly moderated by the COVID-19-related task setbacks.
H6	Psychological resilience moderates the negative and direct relationship between RO and SB such that this relation is less negative (or even positive) at higher psychological resilience than at lower psychological resilience.	Supported	Psychological resilience can significantly mitigate the negative influence of RO on SB, as we expected.
H7	Psychological resilience moderates the positive and indirect relationship between ST and SB (via RO) such that the indirect relationship will be more positive at higher psychological resilience than at lower psychological resilience.	Supported	Psychological resilience is favorable for increasing the performance of ST in SB improvement via reducing RO.

SB = safety behavior, ST = safety training, RO = role overload.

## Data Availability

The data that support the findings of this study are available from the corresponding author upon reasonable request.
